# Stakeholder Perspectives on an Inpatient Hypoglycemia Informatics Alert: Mixed Methods Study

**DOI:** 10.2196/31214

**Published:** 2021-11-26

**Authors:** Nestoras Mathioudakis, Moeen Aboabdo, Mohammed S Abusamaan, Christina Yuan, LaPricia Lewis Boyer, Scott J Pilla, Erica Johnson, Sanjay Desai, Amy Knight, Peter Greene, Sherita H Golden

**Affiliations:** 1 Division of Endocrinology, Diabetes & Metabolism, Department of Medicine, Johns Hopkins University Baltimore, MD United States; 2 Department of Anesthesiology and Critical Care Medicine, Johns Hopkins University Baltimore, MD United States; 3 Division of General Internal Medicine, Department of Medicine, Johns Hopkins University Baltimore, MD United States; 4 Department of Medicine, Johns Hopkins Bayview Medical Center, Johns Hopkins University Baltimore, MD United States; 5 Department of Medicine, Johns Hopkins University Baltimore, MD United States; 6 Department of Cardiac Surgery, Johns Hopkins University Baltimore, MD United States

**Keywords:** informatics alert, clinical decision support, hypoglycemia, hospital, inpatient

## Abstract

**Background:**

Iatrogenic hypoglycemia is a common occurrence among hospitalized patients and is associated with poor clinical outcomes and increased mortality. Clinical decision support systems can be used to reduce the incidence of this potentially avoidable adverse event.

**Objective:**

This study aims to determine the desired features and functionality of a real-time informatics alert to prevent iatrogenic hypoglycemia in a hospital setting.

**Methods:**

Using the Agency for Healthcare Research and Quality Five Rights of Effective Clinical Decision Support Framework, we conducted a mixed methods study using an electronic survey and focus group sessions of hospital-based providers. The goal was to elicit stakeholder input to inform the future development of a real-time informatics alert to target iatrogenic hypoglycemia. In addition to perceptions about the importance of the problem and existing barriers, we sought input regarding the content, format, channel, timing, and recipient for the alert (ie, the *Five Rights*). Thematic analysis of focus group sessions was conducted using deductive and inductive approaches.

**Results:**

A 21-item electronic survey was completed by 102 inpatient-based providers, followed by 2 focus group sessions (6 providers per session). Respondents universally agreed or strongly agreed that inpatient iatrogenic hypoglycemia is an important problem that can be addressed with an informatics alert. Stakeholders expressed a preference for an alert that is nonintrusive, accurate, communicated in near real time to the ordering provider, and provides actionable treatment recommendations. Several electronic medical record tools, including alert indicators in the patient header, glucose management report, and laboratory results section, were deemed acceptable formats for consideration. Concerns regarding alert fatigue were prevalent among both survey respondents and focus group participants.

**Conclusions:**

The design preferences identified in this study will provide the framework needed for an informatics team to develop a prototype alert for pilot testing and evaluation. This alert will help meet the needs of hospital-based clinicians caring for patients with diabetes who are at a high risk of treatment-related hypoglycemia.

## Introduction

### Background

Hypoglycemia is a common occurrence in hospitals and has been linked to poor clinical outcomes and increased mortality [1,2]. Patients with and without diabetes can experience acute hypoglycemic episodes in the hospital, which may result in outcomes ranging from mild distress and patient dissatisfaction with cardiac ischemia, arrhythmias, loss of consciousness, stroke, seizures, and coma [1,3,4]. Of the 8 million and rising number of patients with diabetes who are admitted to hospitals in the United States annually, up to 25% may experience a hypoglycemic episode during hospitalization [5]. Approximately half of these episodes can be explained by an underlying illness, such as severe sepsis, renal failure, liver failure, or malignancy [3]; however, the remaining half are iatrogenic in nature, usually resulting from insulin treatment [6].

Insulin remains the recommended therapy for most patients with diabetes during hospitalization [7,8]. Recent data from the Medicare Patient Safety Monitoring System found that glucose-lowering medications were associated with the highest rates of adverse outcomes for all drugs used in the hospital [9]. As insulin accounts for the vast majority of hypoglycemic events [10,11], the Joint Commission and the Institute for Safe Medication Practices have designated it a *high-alert* medication [12]. Insulin is typically administered as a continuous infusion for critically ill patients in the intensive care unit (ICU) and as subcutaneous injections in noncritically ill patients in general medical or surgical wards. In contrast to the ICU setting where insulin adjustments are driven by nurse-managed protocols, insulin titration in the non-ICU setting is prescriber-driven and requires evaluation of a complex set of clinical, laboratory, and pharmacological parameters.

Clinical decision support (CDS) tools in electronic medical record (EMR) systems have been increasingly used in the United States and have been shown to improve the processes of care and clinical outcomes [13-15]. These tools have been used to alert clinicians, suggest diagnostic or treatment recommendations, and provide contextually pertinent information to optimize care for a wide variety of indications in the hospital setting, ranging from sepsis to acute kidney injury [16-18]. It stands to reason, then, that CDS could be used to improve care of patients vulnerable to hypoglycemia. Prediction models using large EMR data sets have been developed to trigger alerts in patients at risk of hypoglycemia [19-22]. Several studies have found that these predictive models can decrease the rate of inpatient hypoglycemic episodes, with one review finding that it could decrease the incidence of severe hypoglycemia by up to 68% [19,23].

At our institution, we have several existing CDS tools to guide clinicians in selecting a safe and effective initial insulin dosing regimen, including a mandatory subcutaneous insulin order set and an optional insulin CDS tool [24]. Although our subcutaneous insulin CDS tool was derived from evidence-based basal-bolus insulin dosing protocols [25], even universal use of this tool would not be expected to prevent all iatrogenic hypoglycemic events in the hospital because of the impact of acute illness and other clinical factors on glucose homeostasis (eg, nutritional status, renal function, and steroid doses).

### Objectives

To address the need for real-time hypoglycemia risk detection, we recently developed a machine learning algorithm using EMR data that accurately predicts iatrogenic hypoglycemia in rolling 24-hour windows following each blood glucose reading during hospitalization [22]. In planning the translation of this machine algorithm into a real-time informatics alert, we sought to obtain feedback from inpatient clinicians who are responsible for the day-to-day management of blood glucose in the hospital. The objective of this study is to obtain stakeholder input regarding the design of a real-time hypoglycemia informatics alert for use in a hospital setting.

## Methods

### Study Design

This was a mixed methods study that integrated quantitative and qualitative data. The initial stage consisted of an electronic survey (administered in February 2019) that was sent to physicians and advanced practice providers at 2 academic medical centers located in Baltimore, Maryland. The second stage consisted of 2 separate focus groups (conducted in September 2019) with participants recruited from the pool of survey respondents, the goal of which was to expand further on the answers to the survey and identify common themes. This study was approved by the institutional review board at the Johns Hopkins School of Medicine. For the electronic survey, consent was implied from respondent completion, and written informed consent was obtained from all participants in the focus group sessions.

### Survey

To evaluate the key features and functionality of a hypoglycemia risk alert, we developed a 21-item electronic survey that was administered through SurveyMonkey (SVMK Inc) via an embedded email hyperlink (Multimedia Appendix 1). The survey required approximately 7-10 minutes to complete and was sent to hospital-based clinicians involved in glucose management, including medical and surgical residents, hospitalists, surgical advanced practice providers, and inpatient diabetes nurse practitioners (NPs). To encourage participation, the email invitation with the survey link was sent directly by residency program directors (medicine, surgery, neurology, obstetrics/gynecology [OB/GYN]) to all residents in their programs, and by program leaders in the hospitalist, surgical advance practice provider groups, and inpatient diabetes management service. Thus, the total number of recipients who received the survey link (and hence the response rate) was not known by the study investigators.

**Figure 1:**
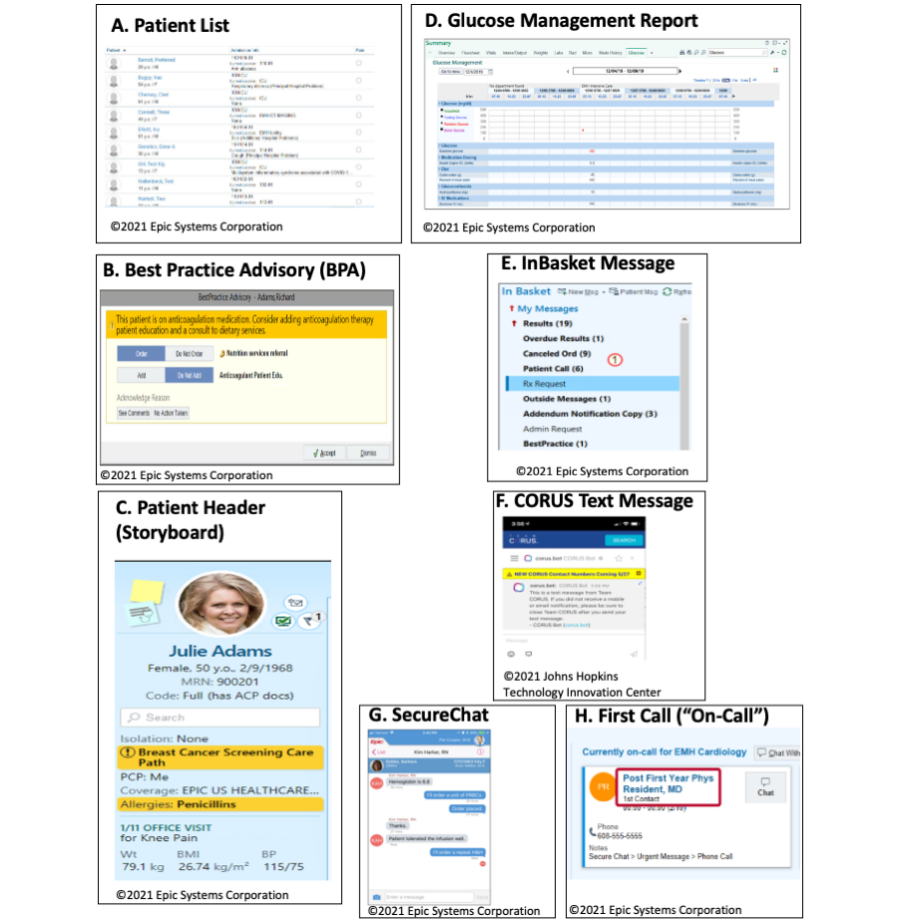
Screenshots examples of proposed formats and channels for informatics alert.

Survey questions were developed using the Agency for Healthcare Research and Quality Five Rights of effective CDS as a guiding framework: the right *information* to the right *person*, in the right interventional *format*, through the right *channel*, and at the right *time* in the workflow [26]. *Right information* refers to the content and presentation of information to the end user (accuracy, estimated risk and reasons for predicted risk, and recommended action). *Right person* refers to the member of the health care team who is most appropriate to receive and respond to the alert and the method of identifying this individual. *Right format* refers to the type of CDS used to address the clinical scenario and *right channel* refers to the platform for communicating the alert. We considered several formats or channels within and outside our EMR system (Epic Version 2018, Epic Systems Corporation), including the best practice advisory (BPA), glucose management print group report, patient system lists, patient header, InBasket messaging, and text messaging. A description of each of these formats or channels is provided in Table 1, with example screenshots in Figure 1. Finally, *right time in the workflow*, a critical component of successful CDS interventions, refers to the time when the alert is presented to the end user in their usual clinical workflow to minimize disruption and achieve desired action. Multimedia Appendix 2 categorizes the survey questions according to the *Five Rights* topics.

**Table 1:** Description of formats and channels considered for alert.

Term	Description	Epic best practice recommendation
Patient list (Figure 1A)	Central hub for clinicians to see patients in their unit and across facility. System list can be compiled across hospital or providers can add a column in their existing patients’ lists to identify patients who meet certain criteria.	Identify populations of patients that clinicians need to review regularly or notify clinicians of individual patients who need their attention.
BPA^a^ (Figure 1B)	Alert that appears based on a wide variety of events and actions.	Use for one-time events that do not recur on a regular basis. Restrict how often and to whom BPAs appear so that they appear only to users who can act on them at a time when they perform the action. Limit the number of BPAs that appear in a separate window.
Patient header (Storyboard; Figure 1C)	Provides patient information relevant to user’s specific role along left side of screen. BPAs can appear in Patient Header so they do not interrupt a clinician’s workflow.	Show information to clinicians that they need to review or that should be available at a glance from anywhere in the chart.
Glucose management report (Figure 1D)	Glucose management report contains summary of all subcutaneous insulin doses over previous 24 hours, and all glucose and insulin doses administered since admission. Allows user to review relevant information about the patient from one spot in EMR^b^ as opposed to searching several areas to compile information.	Use to show information that a clinician needs to make decisions based on the total information compiled in the report.
InBasket message (Figure 1E)	Secure, closed, task-based messaging system to send and receive information about patient care, directly linking messages to patient’s accounts, chart, laboratory results, and orders.	Good fit for questions or issues that do not need to be handled immediately because users might not regularly check all of their InBasket messages.
CORUS text message (Figure 1F)	CORUS: Secure text messaging system developed by Johns Hopkins Technology Innovation Center allowing users to communicate within channels or groups on computer or mobile device. This communication channel is external to EMR.	N/A^c^
Secure chat text message (Figure 1G)	Epic Secure Chat (deployed after completion of this study and will ultimately replace existing CORUS text messaging system) allows users to have conversations with a single recipient or with a group of colleagues securely on a mobile device. This communication channel is internal to EMR.	Intended for quick coordination between members of the team
First Call (Figure 1H)	Designated field to specify on-call provider in EMR.	N/A

^a^BPA: best practice advisory.

^b^EMR: electronic medical record.

^c^N/A: not applicable.

### Focus Groups

To gain a more in-depth understanding of the perceptions, preferences, and perceived barriers to a hypoglycemia informatics alert, we conducted 2 focus group sessions, each consisting of 6 different participants, after the results of the survey were analyzed. Hospital-based physicians, NPs, and PAs were eligible for participation. Participants who responded to the initial survey expressing interest in further research related to the topic were invited to the focus group sessions. No participant identifiers were used during the session, and all participants were referred to by a given letter at the start of the session.

The focus group sessions lasted 60-90 minutes and were led by an experienced focus group moderator (LLB). An institutional review board approved–approved structured interview guide was used (Multimedia Appendix 3), with questions designed to delve into survey results and reasoning behind responses. The principal investigator (NM) was also presented to address or clarify any participant questions during the session. The sessions were audio-recorded and professionally transcribed. The investigators further reviewed and corrected the transcriptional errors. The participants were provided with food during the session and a US $100 gift card.

### Data Analysis

Descriptive statistics were used to summarize the characteristics of the survey respondents and focus group participants. For binary, multiple-choice responses and Likert response data, the number and percentage of responses were provided. For rank items, the average ranking was calculated as the weight of the ranked position. MAXQDA (Verbi Software, 2019) was used for qualitative analysis and thematic coding of data from the focus group sessions. We used a combination of deductive and inductive methods in our analyses [27]. Before coding the data, we identified an initial set of deductive codes centered on the CDS Five Rights framework. Close reading of the transcripts then informed the development of inductive codes, reflecting *the ground*, or participants’ experiences. When the code structure was considered final (ie, no new concepts were apparent), one researcher independently applied the finalized code structure and synthesized the data into patterns (ie, a cohesive category of responses found across our participants) and themes (ie, a broad concept or topic that aggregates patterns).

## Results

### Study Participants

Table 2 shows the characteristics of the survey respondents and participants in the 2 focus group sessions. There were a total of 102 survey respondents, most of whom were internal medicine (IM) physicians, with fairly even representation from trainees and faculty or staff. Focus group 1 comprised 6 NPs (of whom 4 were inpatient diabetes specialists) and Focus group 2 comprised 6 physicians (half of whom were medicine residents).

**Table 2:** Characteristics of survey and focus group respondents.

Characteristic			Survey		Focus group 1		Focus group 2
Participants, n			102		6		6
**Provider type, n (%)**							
	Physician	73 (71.6)		0 (0)		6 (100)	
	Nurse practitioner	21 (20.6)		6 (100)		0 (0)	
	Physician assistant	6 (5.9)		0 (0)		0 (0)	
	Other	2 (1.9)		0 (0)		0 (0)	
**Specialty, n (%)**							
	Medicine	52 (50.9)		0 (0)		3 (50)	
	Surgery	26 (25.5)		2 (33.3)		0 (0)	
	Endocrinology or diabetes	15 (14.7)		4 (66.7)		1 (16.6)	
	Neurology or neurosurgery	5 (4.9)		0 (0)		1 (16.6)	
	Obstetrics/gynecology	3 (2.9)		0 (0)		1 (16.6)	
	Other	1 (0.9)		0 (0)		0 (0)	
**Level of training, n (%)**							
	Resident or fellow	55 (53.9)		0 (0)		6 (100)	
	Faculty or staff	47 (46.1)		6 (100)		0 (0)	
Age (years), mean (SD)			—^a^		39.8 (3.9)		29.5 (2.1)
**Experience (years), n (%)**							
	<5	—		1 (16.6)		6 (100)	
	5-10	—		1 (16.6)		0 (0)	
	≥10	—		4 (66.7)		0 (0)	

^a^Data not collected in the survey.

### Survey Responses

**Figure 2:**
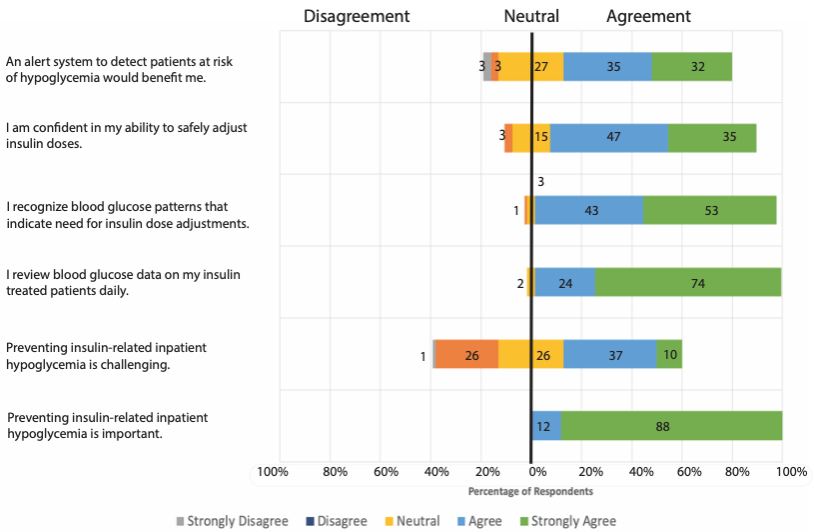
Survey results related to importance of problem and perceived benefit of informatics alert.

Figure 2 summarizes the responses to the survey questions related to the importance of the problem and the perceived benefit of the proposed alert. There was unanimous agreement that preventing insulin-related hypoglycemia in hospitalized patients is an important priority, and 47% (48/102) either agreed or strongly agreed that preventing insulin-related hypoglycemia in the hospital is challenging. Nearly all providers reported that they reviewed blood glucose data on insulin-treated patients and almost all providers felt that they recognized glycemic patterns indicative of a need to adjust insulin doses. Accordingly, 82.3% (84/102) of the respondents felt confident in their ability to safely adjust their insulin doses. Over two-thirds felt that a real-time hypoglycemic alert would be beneficial.

Table 3 summarizes the survey responses related to the proposed information alert. The top 3 preferred formats were an alert indicator in the patient header, secure text message, and alert prompt displayed in the existing glucose management report. The least desired formats were an Epic InBasket message, a patient system list, and a pop-up BPA. In terms of information included in the alert, 78.4% (80/102) of the respondents indicated that they would want to know the specific reasons why an individual patient is predicted to be at risk based on the prediction model; 64.7% (66/102) of the respondents wanted the alert to categorize the predicted hypoglycemia risk into low, medium, and high levels; and 62.8% (64/102) wanted the alert to give an estimated probability of hypoglycemia. A minority of respondents (21/104, 23.5%) indicated that they would want the alert to hyperlink to an actual prediction model, and 16.7% (17/102) indicated that they would want the validated accuracy of the model displayed in the alert. With respect to accuracy, there was a wide range of acceptable sensitivity and specificity thresholds among respondents, with most considering 70% and above to be acceptable for both.

**Table 3:** Survey results.

Survey question		Values
**What is your preferred format for the tool? (average rank, higher score=more desirable)**		
	Patient header	4.59
	Text message	4.03
	Glucose management report	3.81
	BPA^a^	3.65
	Patient list	2.94
	Epic InBasket message	1.98
**What piece of information would you like to see included in the real-time alert? (select all that apply; n=102), n (%)**		
	Specific reason or reasons a patient is at risk based on the prediction model	80 (78.4)
	Categorized risk of hypoglycemia (eg, low, medium, high)	66 (64.7)
	Patients’ estimated probability of hypoglycemia	64 (62.8)
	Hyperlink to actual prediction model	24 (23.5)
	Validated accuracy of the model	17 (16.7)
	None of the above is necessary	3 (2.9)
**What feature would you like to see incorporated in the real-time alert? (select all that apply; n=102), n (%)**		
	Recommended action	90 (88.2)
	Ability to acknowledge the alert	61 (59.8)
	Ability to ignore or override alert	61 (59.8)
	Direct link to subcutaneous insulin order set	54 (52.9)
	Direct link to subcutaneous insulin decision support tool	29 (28.4)
	Ability to consult endocrinology or inpatient diabetes management service	43 (42.2)
	None of the above	1 (0.9)
**Should the real-time informatics alert trigger an endocrinology consult? inpatient diabetes management service consult? (n=101), n (%)**		
	No	85 (84.2)
	Yes	16 (15.8)
**What is your preferred channel for the alert tool? (select all that apply; n=102), n (%)**		
	CORUS text message	63 (61.8)
	BPA tool	52 (50.9)
	Other channels	15 (14.7)
	Epic inBasket message	5 (4.9)
**When would you like to receive the alert in your workflow? (single choice; n=102), n (%)**		
	As soon as hypoglycemia risk is detected	47 (46.1)
	At the same time everyday	27 (26.5)
	When opening the EMR^b^ of a patient predicted to be high risk	24 (23.5)
	Other	4 (3.9)
**Who should receive the alert? (select all that apply; n=102), n (%)**		
	Person listed as *first call* on the EMR	86 (84.3)
	Nurse	47 (46.1)
	Attending physician	15 (14.7)
	Clinical nurse specialist (nurse practitioner)	15 (14.7)
**What is lowest sensitivity you’d consider clinically acceptable for the proposed alert tool? (single choice; n=101), n (%)**		
	50%-59%	3 (2.9)
	60%-69%	10 (9.9)
	70%-79%	37 (36.6)
	80%-89%	37 (36.6)
	90%-100%	14 (13.9)
**What is lowest specificity you’d consider clinically acceptable for the proposed alert tool? (single choice; n=100), n (%)**		
	50%-59%	3 (3)
	60%-69%	6 (6)
	70%-79%	30 (30)
	80%-89%	41 (41)
	90%-100%	20 (2)
**Are you interested in participating in a clinical design team to build the informatics alert? (n=96), n (%)**		
	No	73 (76)
	Yes	23 (24)

^a^BPA: best practice advisory.

^b^EMR: electronic medical record.

The vast majority of the respondents (90/102, 88.2%) indicated that they would want the alert to provide some recommended action, and 59.8% (61/102) of respondents suggested that there should be an option acknowledge the alert or ignore or override the alert. Other features of the alert desired by approximately half or less of the respondents included direct hyperlinks to our subcutaneous insulin order set (54/102, 52.9%) and subcutaneous insulin decision support tool (29/102, 28.4%) and the ability to directly consult endocrinology or inpatient diabetes management service (43/102, 42.2%). Although some respondents felt that the ability to easily consult endocrinology or diabetes services was important, most (85/101, 84.2%) felt that the alert should not automatically trigger a consult.

Regarding the *right person* to receive the alert, 84.3% (86/102) of respondents indicated the person listed as *first call* in the EMR should be notified. In addition to the *first call* provider, 46.1% (47/102) of respondents indicated that the patient’s nurse should also be notified. With respect to the workflow, 46.1% (47/102) of respondents indicated they would want to be notified as soon as the hypoglycemia risk was identified, whereas 26.5% (27/102) preferred the same time every day and 23.5% (24/102) preferred being notified only when entering the chart of a relevant patient.

### Focus Groups

The core themes discussed during the focus groups centered on the CDS five rights format, with the aim of receiving feedback on how a functional alert system could best suit front-link clinicians at a major hospital, as well as anticipated barriers and challenges. Representative quotes illustrating each key theme are presented in Table 4.

**Table 4:** Representative quotes from focus groups.

Theme and pattern		Representative quotes
**Right information**		
	Accuracy	“It’s very important when it’s first deployed that the alert is highly accurate...Because otherwise, I think you risk people developing an attitude that they’re not going to pay attention to it.” [OB/GYN^a^ resident] “I think less accurate alert risks developing provider fatigue.” [OB/GYN resident] “As we get more data over time, you can include something about, like, the probability of a hypoglycemic event in your patient is 53% of all patients with this probability in the past X amount of time. What number or percentage went on to have a hypoglycemic event?” [OB/GYN resident]
	Trigger	“I would want to know why the alert was triggered, initially. It’s not that as a provider that I don’t trust what the computer has calculated to be whatever algorithm that is coming out for my patient, but for my own education and learning.” [OB/GYN resident] “I think having an option that says this alert is inaccurate and not only having that option but that triggering someone to review that alert and see why it was triggered and hopefully, revise it so that it’s more accurate.” [IM^b^ resident]
	Recommended action	“I would say that it would be helpful if the options were there for you to check...if like, you know-like D5 and D10 infusions were there as an option to check to make them an active order.” [Surgical NP^c^] “Maybe putting something like...reduce [insulin dose] by 20% or something that would be helpful.” [Diabetes NP] “One of the things I really appreciate about TREWS [sepsis alert] is that it will guide you through the algorithm and the criteria you need to meet in order to treat the sepsis.” [IM resident]
**Right person**		
	Nurses	“I think the nurse should be one of the first (to be contacted).” [Diabetes NP] “I think the way the TREWS [sepsis alert] is set up is that the nurse gets the first alert and they’re responsible for contacting first call or whoever the primary team is. And so, the benefit of that is they will know how that service is set up whether or not they use the first call.” [IM chief resident]
	First call provider	“First call person...if that’s the updated one.” [Diabetes NP] “First call system and then the BPAs^d^, as long as we can, kind of, minimize it so it’s not—again, the issue with alert fatigue.” [IM resident]
	Consultant	“I’m forever getting alerts from things that are not a consultant team’s responsibility.” [Neurology resident]
**Right format**		
	Laboratory results	“If you can write something under labs. Because I feel like labs, everyone watches.” [Diabetes NP] “Adding a symbol or something to an actual glucose result to say this person is at risk.” [OB/GYN resident]
	Glucose management report	“I think I find that tool to be the one that I use the most when I’m managing a patient’s glucose and insulin because it gives me a good way to review a 24-hour snapshot.” [OB/GYN resident] “I really like the glucose management report. I think for internal medicine, we use it...all the time.” [IM resident] “I think it would be great to have it there. I don’t think it’s mutually exclusive from the other ones, but I will say for an alert, people are not always going to look in there at the right time that you want them to know about the information. This raises the concern that although the tool is made to help specifically with glucose management, that a lack of use by some staff may lead to delay in response to an alert.” [IM chief resident]
	BPA	“BPAs...interrupt your workflow so much.” [IM chief resident]
	Epic InBasket	“Epic InBasket, I agree...It’s useless. Nobody’s going to look there for urgent things.” [IM chief resident] “Especially the EPIC InBasket, we get so many messages every day. Results to follow-up on. It would just get lost. I mean, there’s no way I would ever see that.” [Endocrinology fellow]
	Patient header	“Perhaps there’s something in the header that tells you need to go look at that tab.” [OB/GYN resident] “I think, actually, the header might be because—like with TREWS [sepsis alert], it’s not interfering. It doesn’t, like, get in your face and make you answer something, but it’s there.” [IM resident] “The BPA thing that will show up in the header. It’ll just list that you have BPAs that need to be addressed.” [IM chief resident] “I feel like our patient headers are very crowded, currently, with quite a bit of information. And so, I’m not entirely sure the best format or buildout to make something appear in the header. Maybe it could be something like a symbol that appears that then indicates that you should go look at the glucose management tab.” [OB/GYN resident]
**Right channel**		
	CORUS (text messages)	“All of us, I think, as housestaff, also check CORUS pretty religiously.” [IM resident] “I think in terms of being alerted to it physically as a house staff member, CORUS is the best.” [IM resident]
**Right time in workflow**		
	Real time	“Alerts should be time sensitive and in real time.” [Surgical NP]
	“Snooze” feature	“What do you think about the idea of—you know, when your computer lets you know there are updates and it needs to be restarted and you say, ‘Not now. Try again in an hour. Not now. Try again tonight.’ I’m saying this alert comes up—I’m opening a chart for a specific reason. I have a task or multiple tasks in mind.” [OB/GYN resident] “Or if there was a way for it to be paused and then it pops up again when you go to click out of the chart. It’s like, hey, don’t forget—you’ve got this thing to do.” [Neurology resident]
	Disruption	“Oftentimes, you’ll just click something to get it out of the way, do what you’re doing, and then you’ll forget about it afterward.” [IM chief resident] “Which I think would be helpful to, like-I need to put in another order right now that’s actually more urgent for the patient, believe it or not. And I don’t want to forget to do that, because I’m messing around now in their Lantus [insulin] dosing, and there and then I forget...or I now have gotten into this big rabbit hole of 6 different orders that I have to place in order to put in the original order that I wanted to put in.” [Surgical NP]
**Barriers or challenges**		
	Importance of problem	“I think the good question to ask is whether or not we have enough of a problem in which patients get into real trouble, as opposed to just having a glass of orange juice.” [Surgical NP] “I think providers are more reactive to hyperglycemia than they are hypoglycemia.” [Diabetes NP] “And that’s rare, right? 99% of these patients are treated with a glass of juice.” [Surgical NP]
	Communication	“Plus, there are so many other providers involved when you order something, or you recommended something.” [Diabetes NP]
	Provider factors	“When they see the blood sugar is high, [providers]...keep giving insulin without understanding the duration of action of the insulin. So, the patient ends up getting stacked.” [Diabetes NP]
	Patient factors	“It’s impossible for us to walk in and put the tray down in front of them, check their sugar, give them insulin, and then they are guaranteed to eat >50% of the tray in front of them.” [Surgical NP] “How much different a diet, in particular, relates to this. People would be on like 100 units of insulin a day at home and if you put them on that [amount of insulin] here, [their blood glucose] will shoot to 0.” [IM resident] “Our patients are non-compliant people, and so, they come in and their home regimen has been ramped up in the outpatient setting because they just aren’t doing it and then you’re trying to guess, sort of, like what are your actual insulin needs and you either become too conscientious and they’re entirely way too hyperglycemic or we’re not conscientious enough in trying to guess, sort of, what’s their appropriate doses. It’s really challenging.” [Neurology resident]
	Alert fatigue	“As a consulting physician, to be honest with you, I don’t really even look at them. I just kind of click to get it out of my way button...I’m not the one who actually has to deal with it 90% of the time.” [Endocrinology fellow] “What drives me most crazy is, if you’ve already answered the questions and then the next time you log into Epic, it shows up again, and again, and again, and again.” [Surgical NP] “Then I know we have the new hypoglycemia alerts that pop up, but I think they pop up very, very, very frequently to the point that I think it’s almost starting to cause a little bit of fatigue.” [IM resident]

^a^OB/GYN: obstetrics/gynecology.

^b^IM: internal medicine.

^c^NP: nurse practitioner.

^d^BPA: best practice advisory.

### Right Information

Accuracy was a common theme. According to the surveys, most of our participants wanted the alert to be at the very least 70% sensitive and specific. One participant noted as follows:

It’s very important when it’s first deployed that the alert is highly accurate...Because otherwise, I think you risk people developing an attitude that they’re not going to pay attention to it.

OB/GYN resident

Participants raised concerns that if the specificity was low at launch, the alert would go off repeatedly and would be ignored. Many noted that similar alert systems have been deployed at our institution before validation of accuracy; as a result, many of the alerts were initially unsuccessful at gaining end user buy-in and changing behavior. To avoid this, one participant noted that the algorithm should be continuously adjusted to increase accuracy over time.

A common theme among focus group participants was the importance of defining specific reasons that triggered an alert. Participants felt that sharing this information would present a learning opportunity for end users and could indirectly modify behavior in their care of future patients. Participants also voiced the concern that there should be an option to report the alert if it is inaccurate:

I think having an option that says this alert is inaccurate and not only having that option but that triggering someone to review that alert and see why it was triggered and hopefully, revise it so that it’s more accurate.

IM resident

Finally, echoing the results of our survey, the focus group participants emphasized their desire to have an alert to recommend the appropriate action. The tendency of alert systems to interrupt workflow without recommending an appropriate action leads to clinicians feeling overwhelmed. Participants noted that the best part of the other alert systems was the ability to help with workup and management. One participant noted as follows:

One of the things I really appreciate about TREWS [sepsis alert] is that it will guide you through the algorithm and the criteria you need to meet in order to treat the sepsis.

IM resident

### Right Person

There were mixed opinions among the focus group participants as to the right person to be notified of the alert. Many believed that contacting the individual or service listed as *first call* in the EMR would be the most sensible approach. However, many voiced the concern that the only way a first-call system would be efficient was if the EMR was accurately updated, which may not always be the case. Other participants voiced that the right person to be alerted should always be a nurse. One participant noted that with other alert systems, the nurse is responsible for triaging the alert and determining whether contacting the provider is required:

I think the way the TREWS [sepsis alert] is set up is that the nurse gets the first alert and they’re responsible for contacting first call or whoever the primary team is. And so, the benefit of that is they will know how that service is set up whether or not they use the first call.

IM resident

### Right Format

There was substantial heterogeneity in preferences regarding the alert format, and our discussion focused on the pros and cons of each format. Focus group participants noted that an alert indicator in the patient header would be a very suitable place for a hypoglycemia alert; however, concerns were raised that the patient header is already a very crowded space:

I feel like our patient headers are very crowded, currently, with quite a bit of information. And so, I’m not entirely sure the best format or buildout to make something appear in the header.

OB/GYN resident

Others really valued the idea of using the glucose management report, which is a tabular report summarizing insulin and glucose data in a temporal way that facilitates pattern recognition and insulin dose adjustments but cautioned that this passive approach may not achieve the desired action. A suggestion raised during the focus groups session that was not considered in the electronic survey was made to place an alert symbol next to a patient’s glucose laboratory result to notify them of impending risk; this alert would be distinct from an abnormal laboratory result value to distinguish predicted risk from overt hypoglycemia. Participants felt that a *results flag* would be the most effective in increasing situational awareness:

If you can write something under labs...because I feel like labs, everyone watches.

Diabetes NP

A consensus was also reached that BPAs were not ideal for an alert system, as they were noted to be very interruptive by some of the participants:

BPAs...interrupt your workflow so much.

IM resident

The Epic InBasket format was dismissed by most participants, as this is not a format routinely used for communication in the inpatient setting:

Epic InBasket, I agree...It’s useless. Nobody’s going to look there for urgent things.

IM chief resident

### Right Channel

The CORUS text messaging system was the unanimously desired channel for receiving the alert. All participants agreed that it was the quickest and most efficient way to be notified. A few even mentioned that they were too heavily reliant on CORUS, but noted it was the appropriate app to get the staff’s attention:

All of us...as housestaff also check CORUS pretty religiously.

IM resident

### Right Timing

The majority agreed that the alert should be direct and in real time to allow for appropriate action. One concern that many had was the fact that the alert might go off in a time when the staff member could not respond. One participant offered the suggestion of a *snooze button* to address this concern:

What do you think about the idea of—you know, when your computer lets you know there are updates and it needs to be restarted and you say, “Not now. Try again in an hour. Not now. Try again tonight.” I’m saying this alert comes up...I’m opening a chart for a specific reason. I have a task or multiple tasks in mind.

OB/GYN resident

### Barriers to Alert Systems

The focus group sessions elicited feedback about barriers and challenges related to hypoglycemic prevention and the proposed informatics alert. Some of the themes that emerged were: (1) iatrogenic hypoglycemia is not serious enough to justify an alert, (2) patient factors that cannot be controlled (eg, snacking without knowledge of the treating team or resistance to taking recommended insulin doses), (3) provider factors, including knowledge gaps, and, most importantly, (4) concerns regarding alert fatigue. Although the survey respondents almost unanimously agreed that preventing hypoglycemia was an important problem, some of the focus group participants seemed to question its significance. For example, one participant commented as follows:

I think the good question to ask is whether or not we have enough of a problem in which patients get into real trouble, as opposed to just [needing to treat with] a glass of orange juice.

Surgical NP

Many participants voiced concern that the alert system must be structured to prevent alert fatigue. Participants noted that other alert systems were constantly popping up inappropriately leading to *people clicking through them* and interrupting the workflow.

## Discussion

### Principal Findings

Overall, the findings from this study indicate that iatrogenic hypoglycemia was perceived as an important problem that could potentially be addressed with a real-time informatics alert. We identified several key design requirements and preferences for the alert: (1) sufficiently accurate, (2) recommendations for care, (3) nondisruptive, and (4) communicated in near real-time to the responsible clinician. Several EMR tools, including an alert indicator embedded in the patient header, glucose management report, and flagged laboratory results, were deemed acceptable formats for consideration of hypoglycemia alerts. Although clinicians indicated that pop-up (interruptive) BPAs would be considered unacceptable because of alert fatigue and disruption of clinical workflow, we were surprised by the acceptability of the use of the hospital’s text messaging system as a channel of communication for the alert because real-time text messaging could be interrupted.

### Comparison With Prior Work

To our knowledge, there are no previous studies soliciting stakeholder input regarding inpatient hypoglycemia informatics alerts. A systematic review and meta-analysis of computerized CDS systems (CDSS) found that push notifications, the ability to execute action, and an interruptive element are the most prevalent features of CDSS that have been evaluated in controlled clinical trials [14]. Absolute incremental improvements in clinical processes or outcomes were demonstrated for CDSS that offered the ability to execute the desired action, and a trend toward improvement was shown for those that allowed for acknowledgment, were behavior-targeted, embedded in the EMR, and interrupted [14].

We recently developed a machine learning algorithm using EMR data that would be considered sufficiently accurate (sensitivity and specificity of approximately 80% and c-statistic of 0.90) based on the input received from stakeholders in this study [22]. As we progress toward translating our machine learning model into a real-time alert, we will seek to consider the key findings from this study to optimize end user buy-in and adoption. Specifically, we intend to develop a near real-time alert that provides specific and actionable recommendations to the ordering provider without disrupting their clinical workflow. The top candidate CDS formats we will consider based on the results of this study are a nonintrusive BPA embedded in the patient header, laboratory result, or existing glucose management report. The selection of the final CDS format will depend in part on our institutional requirements and programing feasibility.

### Limitations

It is important to note that there have been three key system changes at our institution since we completed this study, which may have affected our findings. First, our text messaging system (CORUS) has been replaced by an alternative text messaging platform that resides within the EMR and is accessible through mobile devices (SecureChat). The main difference in the text messaging platform change is that the communication resides within the EMR and is accessible via a mobile app. Communications delivered via SecureChat would allow the user to quickly access other relevant information within the patient’s chart without the need to access an external system. Second, a new feature called *Storyboard* was released in an Epic software upgrade, which shifted the patient header information from the top of the chart to the left side of the screen. The Storyboard includes patient identifiers and key information, including allergies, infection or isolation, and best practice advisories. At our institution, the Storyboard is already being used to summarize active alerts for a given patient (eg, sepsis) and would be a potential consideration for the development of hypoglycemia alerts. We believe that the inferences of this study related to the patient header would apply to the new Storyboard format. A third system change at our institution has been made in which all services are required to either list an individual or a service pager name (for surgical services) in the first call field. It is expected that the first call field will serve as the source of truth in identifying the responsible provider for a given patient at any given time.

### Conclusions

Alert fatigue is a commonly cited limitation in CDSS [28-30]. Despite the fact that avoidance of alert fatigue emerged as one of the most important themes in our analysis, some incongruencies were identified among participants in this study. On the one hand, clinicians strongly opposed a pop-up style BPA; on the other hand, real-time alerting via text messaging was identified as a preferred channel of communication regarding hypoglycemia risk. Although a text message can perhaps be more easily ignored than a pop-up BPA, both forms of alerting could distract the clinician from their current clinical work. On the basis of the overall input we received from our stakeholders, we place greater value on avoidance of alert fatigue and disruption in clinical workflow than immediate notification of the risk of hypoglycemia. Therefore, we will explore several strategies to minimize alert fatigue, such as communicating increased risk in a nonintrusive fashion in the patient header, laboratory result section, or glucose management report.

Future research will assemble an informatics design team to develop prototypes using the most desired formats proposed by stakeholders in this study, and to test these prototypes in a pilot observational study before being widely implemented and evaluated for effectiveness. Ensuring that the alert system follows the CDS Five Rights and adheres and aligns with stakeholder preferences from this study will hopefully improve the usability, adherence, and efficacy of the hypoglycemic alert.

## Appendix

Multimedia Appendix 1 Survey question response types and concepts.

Multimedia Appendix 2 Electronic survey.

Multimedia Appendix 3 Structured interview guide.

## Abbreviations

BPA: best practice advisory
CDS: clinical decision support
CDSS: clinical decision support system
EMR: electronic medical record
ICU: intensive care unit
IM: internal medicine
NP: nurse practitioner
OB/GYN: obstetrics/gynecology
