# Supplementary material for: Stakeholder Perspectives on an Inpatient Hypoglycemia Informatics Alert: Mixed Methods Study
Source: JMIR Hum Factors. 2021 Nov 26;8(4):e31214. doi: 10.2196/31214 (PMC8665392; doi:10.2196/31214)
Supplement: Multimedia Appendix 1 [file humanfactors_v8i4e31214_app1.docx]

## Supplemental Table 1. Survey Question Response Types and Concepts

|  |  |  | **Concept** | | | | | |
| --- | --- | --- | --- | --- | --- | --- | --- | --- |
| **Question No.** | **Topic** | **Response type** | **Right information** | **Right person** | **Right format** | **Right channel** | **Right Time in Workflow** | **Other** |
| 1 | Provider Type | Multiple choice (single answer) |  |  |  |  |  | x |
| 2 | Specialty | Multiple choice (single answer) |  |  |  |  |  | x |
| 3 | Level of training | Yes/No |  |  |  |  |  | x |
| 4 | Manage glucose in hospital | Yes/No |  |  |  |  |  | x |
| 5 | Importance of problem | Likert scale |  |  |  |  |  | x |
| 6 | Challenge managing glucose |  |  |  |  |  |  | x |
| 7 | Daily review of blood glucose data |  |  |  |  |  | x |  |
| 8 | Recognizing glucose patterns |  | x |  |  |  |  |  |
| 9 | Confidence in adjusting doses |  | x | x |  |  |  | x |
| 10 | Usefulness of alerts as clinical decision support |  | x |  | x |  |  |  |
| 11 | Proposed alert format | Rank 6-items |  |  | x |  |  |  |
| 12 | Desired information | Multiple choice (select all) | x |  |  |  |  |  |
| 13 | Desired alert features | Multiple choice (select all) | x | x |  |  | x |  |
| 14 | Automated inpatient diabetes consult | Yes/No |  | x |  | x |  |  |
| 15 | Preferred channel of alert | Multiple choice (select all) |  |  |  | x |  |  |
| 16 | Timing of alert in workflow | Multiple choice (single answer) |  |  |  |  | x |  |
| 17 | Who should receive alert | Multiple choice (select all) |  | x |  |  |  |  |
| 18 | Desired sensitivity of alert | Multiple choice (single answer) | x |  |  |  |  |  |
| 19 | Desired specificity of alert | Multiple choice (single answer) | x |  |  |  |  |  |
| 20 | Comments | Free-text response |  |  |  |  |  | x |
| 21 | Willingness to participate in focus group or clinical design team | Yes/No |  |  |  |  |  | x |
