# Supplementary figures and images for: Stakeholder Perspectives on an Inpatient Hypoglycemia Informatics Alert: Mixed Methods Study
Source: JMIR Hum Factors. 2021 Nov 26;8(4):e31214. doi: 10.2196/31214 (PMC8665392; doi:10.2196/31214)

## Multimedia Appendix 2. Electronic Survey.


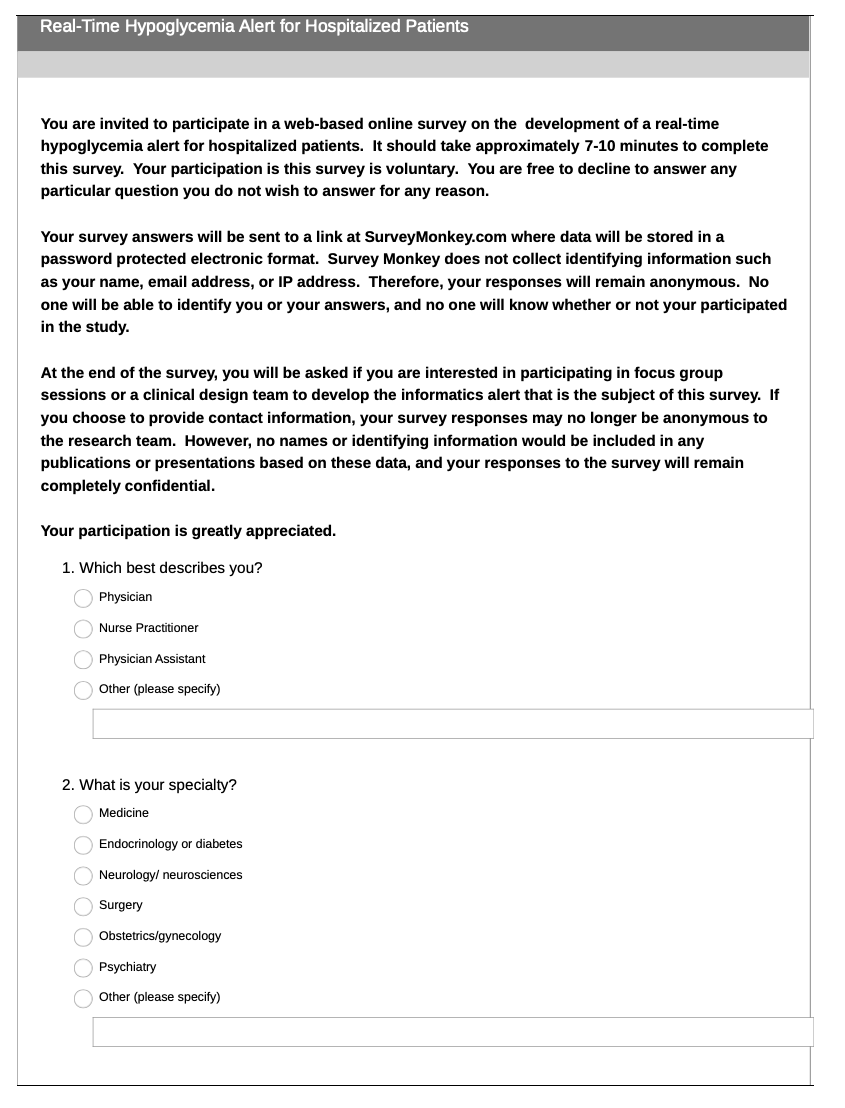


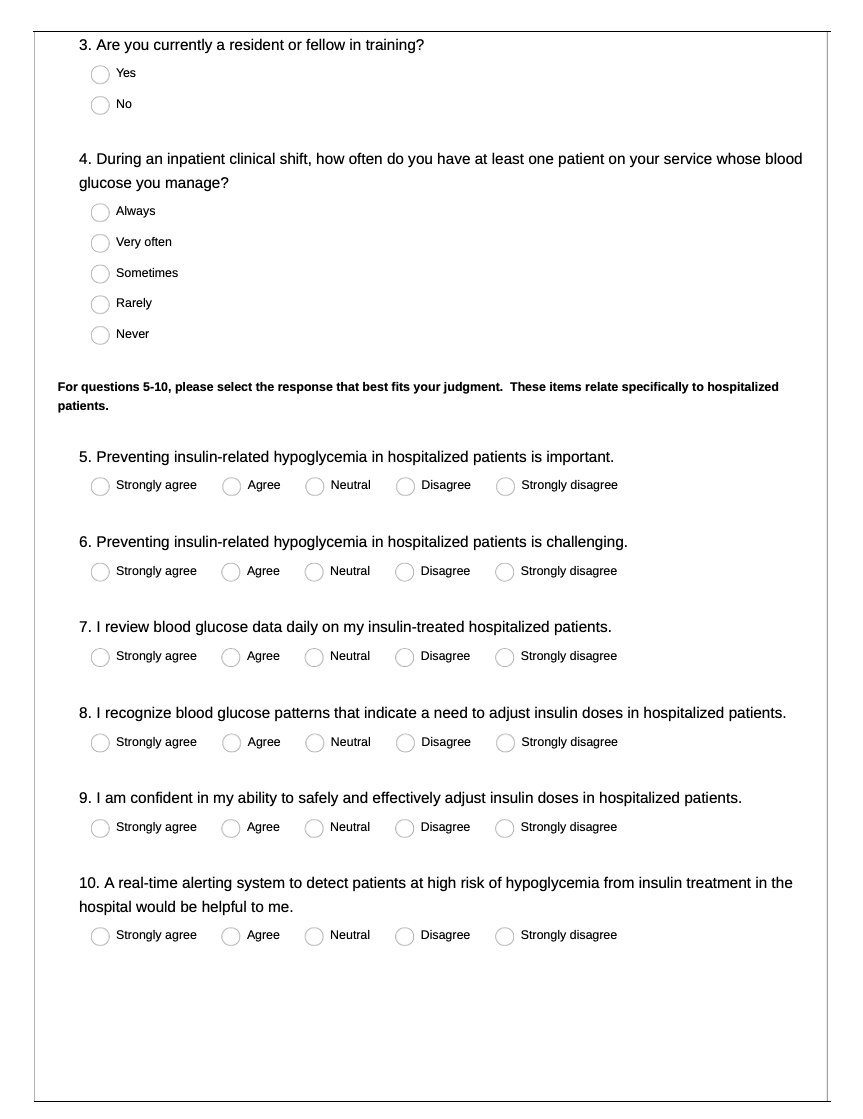


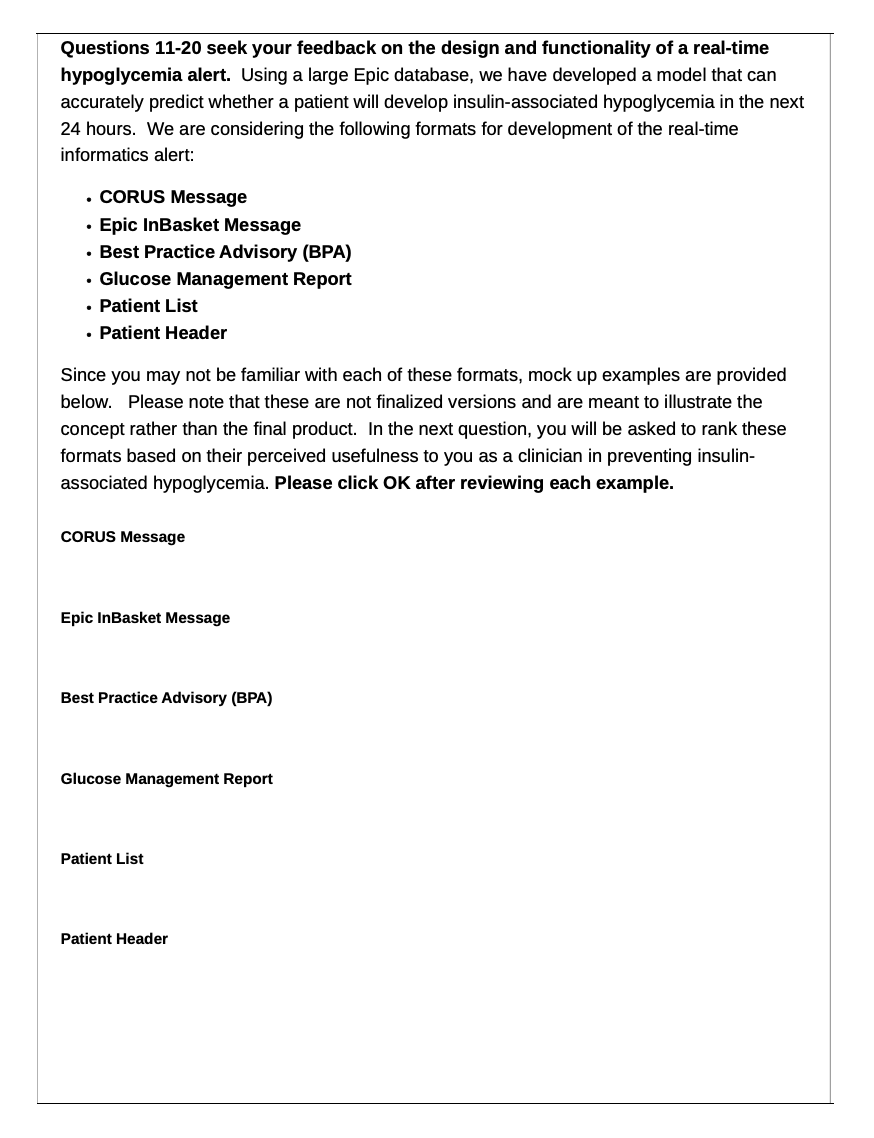


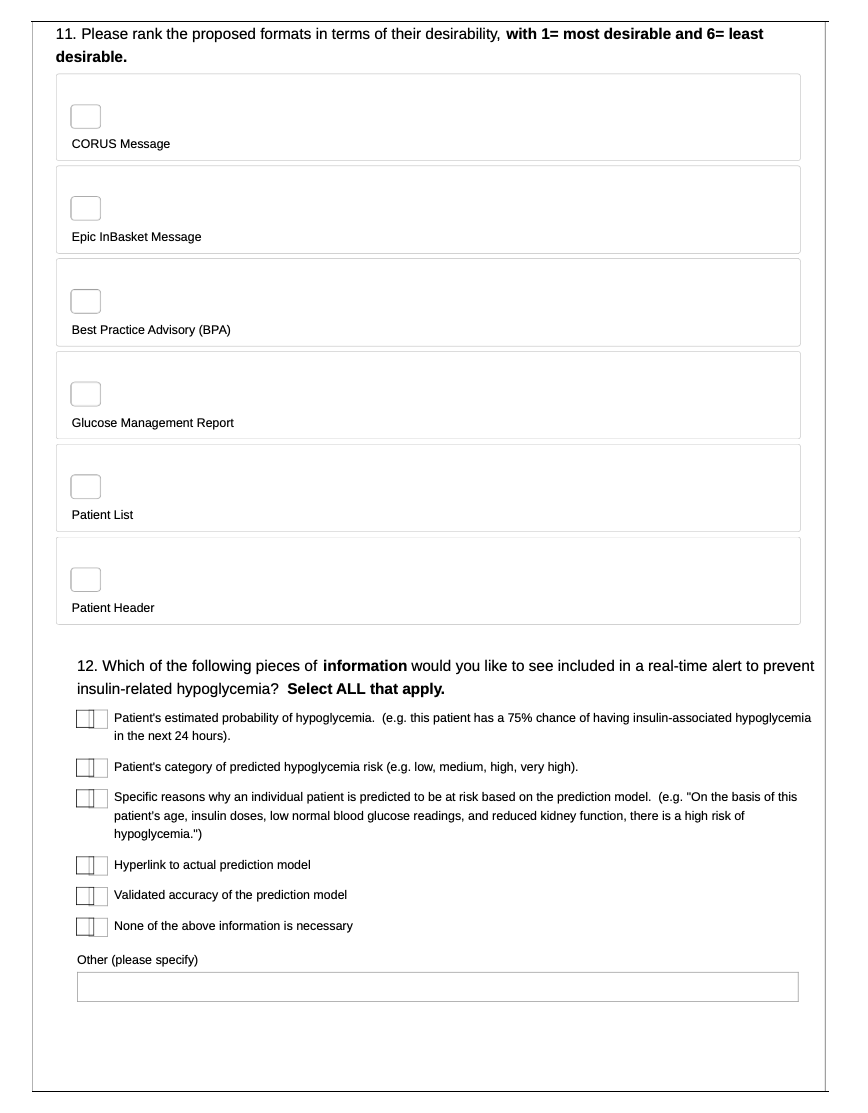


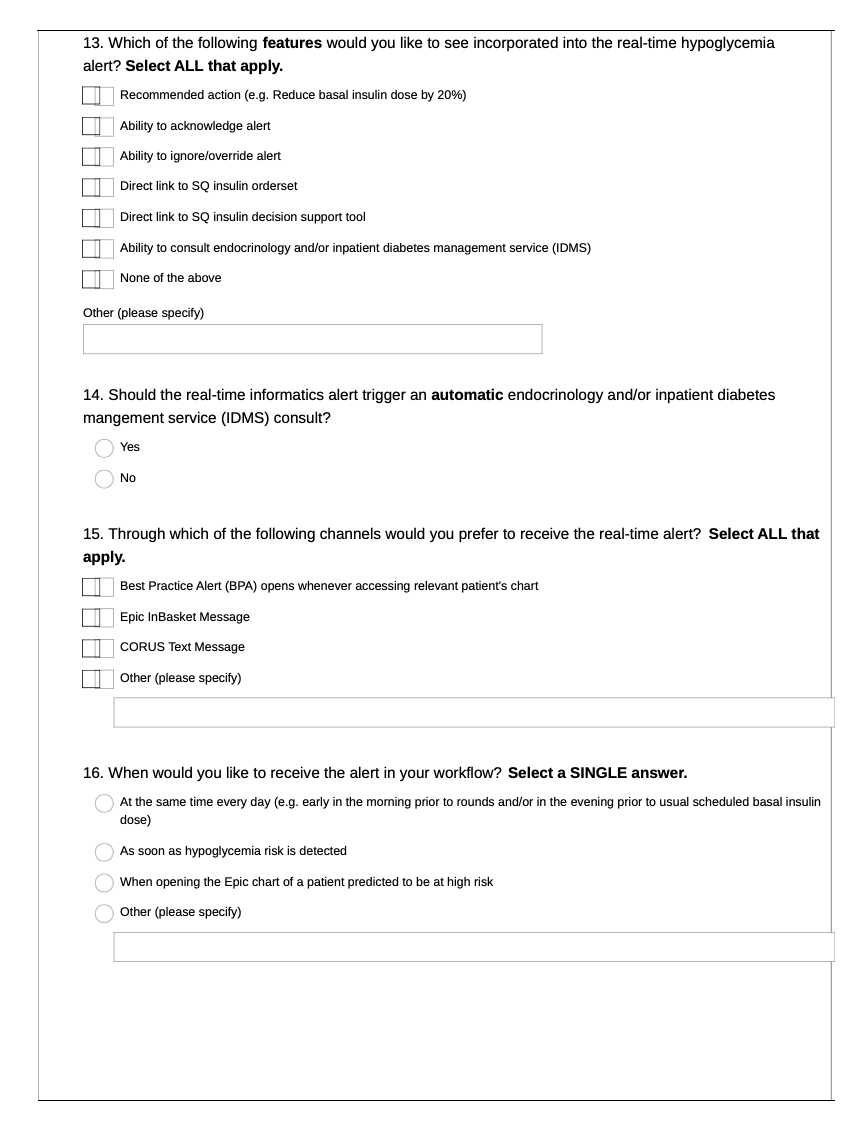


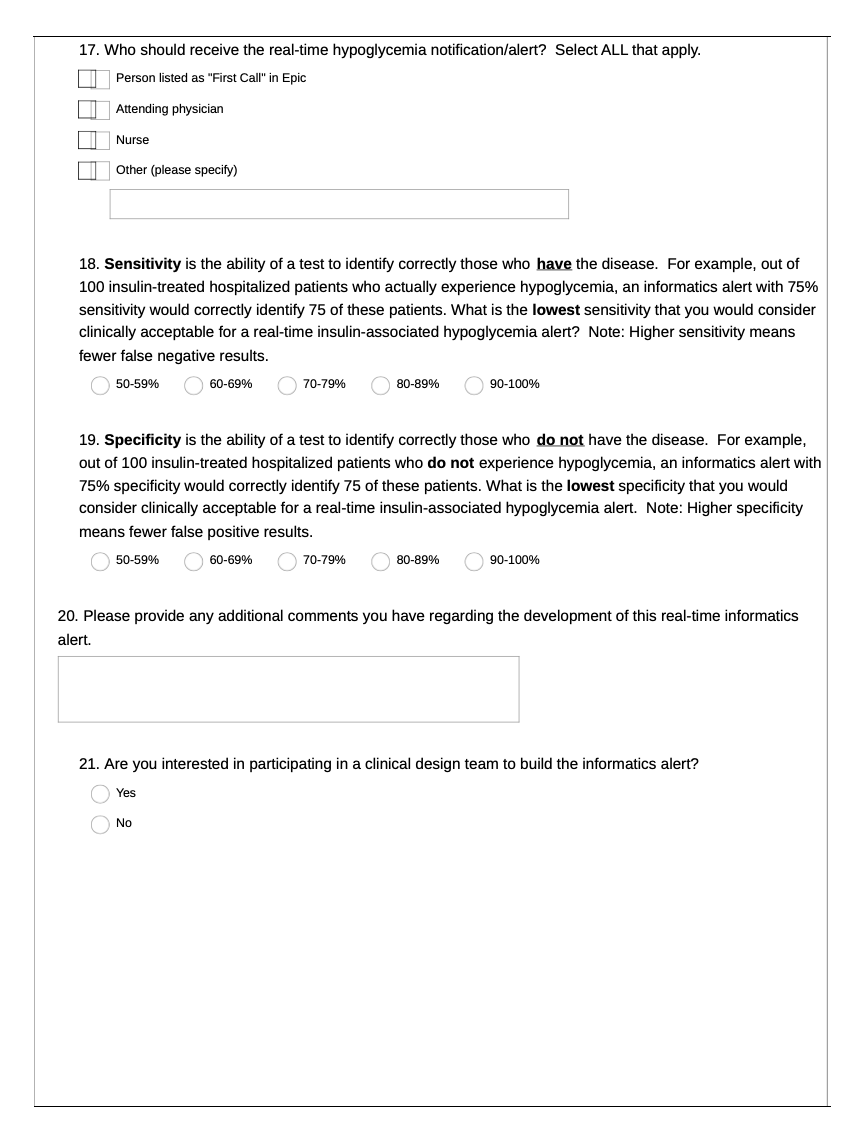


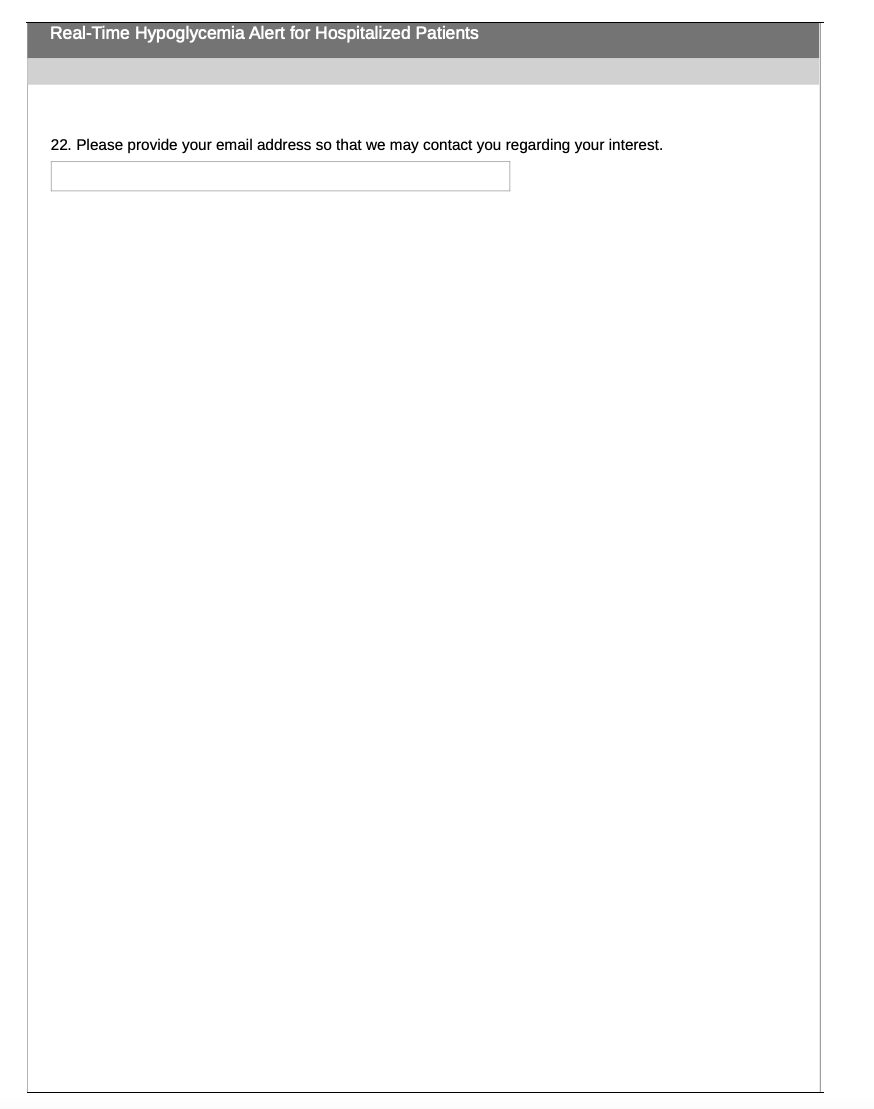

Supplement: Multimedia Appendix 2 [file humanfactors_v8i4e31214_app2.docx]
