# Supplementary material for: Stakeholder Perspectives on an Inpatient Hypoglycemia Informatics Alert: Mixed Methods Study
Source: JMIR Hum Factors. 2021 Nov 26;8(4):e31214. doi: 10.2196/31214 (PMC8665392; doi:10.2196/31214)
Supplement: Multimedia Appendix 3 [file humanfactors_v8i4e31214_app3.docx]

## Multimedia Appendix 3. Structured Interview Guide

Good afternoon and welcome to our session. Thank you for taking the time to join us to share your opinions regarding the development of a real-time informatics alert to prevent insulin-related hypoglycemia in the hospital. My name is LaPricia Boyer and I will be moderating this session together with Dr. Mathioudakis, who is the PI on this study.

You were invited to this session because you are a clinician who works in the hospital setting and manages blood glucose for your hospitalized patients. We would like to know your opinions regarding insulin-related hypoglycemia so that we can develop a solution to this problem that will be useful to you as a clinician who practices in the hospital.

Just some ground rules before we start. There are no wrong answers but rather differing points of view. Please feel free to share your point of view even if it differs from what others have said. Keep in mind that we’re interested in negative comments as well as positive comments, and at times the negative comments are the most helpful.

You’ve probably noticed the microphone. We’re tape recording the session because we don’t want to miss any of your comments. People often say very helpful things in these discussions and we can’t write fast enough to get them all down. To maintain your anonymity, we will ask that you state the letter on your card in front of you before you speak. We won’t use any names in our reports. You may be assured of complete confidentiality. Nothing that you say here today will be shared with your supervisor or program directors.

In the spring, we sent out an electronically questionnaire via Survey Monkey on the topic that is the focus of today’s discussion and we received responses from 102 clinicians. The information we received was very useful and will help guide our discussion today. We’d like to delve deeper into some of the questions that were asked in the questionnaire that we are sure we have a clear understanding of what factors are influencing the responses. In some of the questions we will ask today, we will reference the results of our questionnaire and we would like your opinions about the reasons why clinicians may have responded the way the did. A hard copy of the anonymized results is in front of you. Whenever we are referencing one of these questions, we will direct you to the specific question in the survey so that you can see how people responded.

Well, let’s begin. If we could start by going around the room and introducing one another. You do not need to say your name, but if you could please tell what specialty you are in (for example, internal medicine, surgery, neurology).

**Questions:**

1. What are the most common alerts that you are familiar with in Epic, particularly in the inpatient setting?
2. How do you feel about informatics alerts, like BPAs, in Epic?
3. We are interested in creating an alert that will fire when a patient is predicted to be at risk from insulin-related hypoglycemia in the next 24 hours. How do you feel about our idea of developing this type of alert?
4. I’d like to direct your attention to question #11 in the survey, which can be found on page 11. This question relates to the desired format of the alert. Clinician respondents to our questionnaire indicated that their desired formats for a hypoglycemia alert, from most useful to least useful, are Patient Header, CORUS message, Glucose Management Report, Best Practice Advisory, Patient List, Epic InBasket Message. Please note that a higher score indicates a more desirable format. What experiences do you have with alerts using any of these formats in the hospital? What are some of the advantages or disadvantages you can think of with using these formats? Which one would you prefer for a hypoglycemia alert?
5. One format we are considering for the hypoglycemia alert is similar to the TREWS notification for sepsis that was recently deployed. How do you feel about the TREWS (sepsis) alert? What do you think about modeling the hypoglycemia notification around the TREWS notification?
6. I’d like to direct your attention to question #15 in the survey, which can be found on page 15. This question relates to the preferred channel of communicating the alert. Clinician respondents indicated that CORUS text messages would be the preferred channel of receiving the alert, followed by a BPA, which could open whenever accessing the relevant patient’s chart. How do you feel about receiving alerts via CORUS messages? What are the advantages and disadvantages of using these channels of communication for a hypoglycemia alert?
7. I’d like to direct your attention to question #17 in the survey, which can be found on page 17. This question relates to which healthcare team member should receive the real-time notification. Clinician respondents indicated that the person listed as “first call” in Epic should be preferred. What are your experiences with the first call designation in Epic? Do you find that this a reliable way of designation who is responsible for the patient? Clinicians also selected the nurse as the primary contact person for the alert. What are your thoughts about this? What are the advantages and disadvantages of directing the notification to nurses?
8. What type of information do you think should be included in the alert?
9. How accurate do you think the alert needs to be in order to be useful?
10. A common theme that emerged from the clinician survey was alert fatigue. Can you share any experiences related to alert fatigue in the hospital. Can you think of ways to minimize alert fatigue?
11. Please share with us any other thoughts you may have that we have not already covered.
